# Combination of Peri-Tumoral and Intra-Tumoral Radiomic Features on Bi-Parametric MRI Accurately Stratifies Prostate Cancer Risk: A Multi-Site Study

**DOI:** 10.3390/cancers12082200

**Published:** 2020-08-06

**Authors:** Ahmad Algohary, Rakesh Shiradkar, Shivani Pahwa, Andrei Purysko, Sadhna Verma, Daniel Moses, Ronald Shnier, Anne-Maree Haynes, Warick Delprado, James Thompson, Sreeharsha Tirumani, Amr Mahran, Ardeshir R Rastinehad, Lee Ponsky, Phillip D. Stricker, Anant Madabhushi

**Affiliations:** 1 Department of Biomedical Engineering, Case Western Reserve University, Cleveland, OH 44106, USA; rakesh.shiradkar@case.edu (R.S.); axm788@case.edu (A.M.); 2 Department of Radiology, Case Western Reserve University, Cleveland, OH 44106, USA; shivani.pahwa@case.edu; 3 Section of Abdominal Imaging and Nuclear Radiology Department, Cleveland Clinic, OH 44195, USA; puryska@ccf.org; 4 Department of Radiology, College of Medicine, University of Cincinnati, Cincinnati, OH 45221, USA; drsadhnaverma@gmail.com; 5 Department of Medicine, University of New South Wales, Sydney, NSW 2052, Australia; daniel.moses@unsw.edu.au (D.M.); ronshnier@gmail.com (R.S.); jjthomo@hotmail.com (J.T.); phillip@stricker.com.au (P.D.S.); 6 Cancer Division, The Kinghorn Cancer Centre/Garvan Institute of Medical Research, NSW 2010, Australia; a.haynes@garvan.org.au; 7 Douglass Hanly Moir Pathology, Sydney, NSW 2000, Australia; wdelprado@dhm.com.au; 8 Garvan Institute of Medical Research, Sydney, NSW 2010, Australia; 9 Urology Institute, University Hospitals Cleveland Medical Center, Case Western Reserve University, Cleveland, OH 44106, USA; Sreeharsha.Tirumani@UHhospitals.org (S.T.); amr.mahran@uhhospitals.org (A.M.); Lee.Ponsky@uhhospitals.org (L.P.); 10 Urology at Lenox Hill and Northwell Health, New York, NY 10075, USA; arastine@northwell.edu; 11 Department of Urology, St. Vincent’s Clinic, Sydney, NSW 2010, Australia; 12 Louis Stokes Cleveland VA Medical Center, Cleveland, OH 44106, USA

**Keywords:** radiomics, prostate cancer, MRI, artificial intelligence, PIRADS, machine learning, peritumoral region

## Abstract

*Background:* Prostate cancer (PCa) influences its surrounding habitat, which tends to manifest as different phenotypic appearances on magnetic resonance imaging (MRI). This region surrounding the PCa lesion, or the peri-tumoral region, may encode useful information that can complement intra-tumoral information to enable better risk stratification. *Purpose*: To evaluate the role of peri-tumoral radiomic features on bi-parametric MRI (T2-weighted and Diffusion-weighted) to distinguish PCa risk categories as defined by D’Amico Risk Classification System. *Materials and Methods*: We studied a retrospective, HIPAA-compliant, 4-institution cohort of 231 PCa patients (*n* = 301 lesions) who underwent 3T multi-parametric MRI prior to biopsy. PCa regions of interest (ROIs) were delineated on MRI by experienced radiologists following which peri-tumoral ROIs were defined. Radiomic features were extracted within the intra- and peri-tumoral ROIs. Radiomic features differentiating low-risk from: (1) high-risk (L-vs.-H), and (2) (intermediate- and high-risk (L-vs.-I + H)) lesions were identified. Using a multi-institutional training cohort of 151 lesions (D1, *N =* 116 patients), machine learning classifiers were trained using peri- and intra-tumoral features individually and in combination. The remaining 150 lesions (D2, *N =* 115 patients) were used for independent hold-out validation and were evaluated using Receiver Operating Characteristic (ROC) analysis and compared with PI-RADS v2 scores. *Results*: Validation on D2 using peri-tumoral radiomics alone resulted in areas under the ROC curve (AUCs) of 0.84 and 0.73 for the L-vs.-H and L-vs.-I + H classifications, respectively. The best combination of intra- and peri-tumoral features resulted in AUCs of 0.87 and 0.75 for the L-vs.-H and L-vs.-I + H classifications, respectively. This combination improved the risk stratification results by 3–6% compared to intra-tumoral features alone. Our radiomics-based model resulted in a 53% accuracy in differentiating L-vs.-H compared to PI-RADS v2 (48%), on the validation set. *Conclusion*: Our findings suggest that peri-tumoral radiomic features derived from prostate bi-parametric MRI add independent predictive value to intra-tumoral radiomic features for PCa risk assessment.

## 1. Introduction

Prostate cancer (PCa) is the second most common cancer in American men with an estimated 191,930 new diagnoses in 2020 [1]. Treatment strategy for PCa patients is determined based on their risk of progression. The D’Amico Risk Classification System (DRCS) is widely used to assess PCa risk of progression based on clinical parameters (initial serum prostate-specific antigen (PSA), T-stage from digital rectal examination (DRE), and biopsy Gleason Score) and is often one of the key criteria in identifying patients who might benefit from radical therapy versus active surveillance [2].

Multi-parametric magnetic resonance imaging (mpMRI) is being used more frequently in PCa detection and characterization [3,4,5]. The Prostate Imaging and Reporting Data Standard (PI-RADS) scheme has been established to streamline the process of identifying clinically significant PCa [6,7]. However, PI-RADS evaluations have been found to significantly vary depending on the radiologist’s experience where inter-reader disagreement is often observed [7,8].

Radiomics [9,10], or the process of computationally extracting features from radiographic images for quantitatively characterizing disease patterns, has been used for PCa risk stratification [11], predicting Gleason score (GS) of the lesion [12], and treatment outcome [13]. These approaches typically involve textural analysis of the PCa lesion (intra-tumoral (IT)) [14]. Bi-parametric MRI (T2-weighted (T2W) and diffusion-weighted (DWI) MRI) has been shown to be advantageous compared to mpMRI [15] and is also being used in radiomic analysis with promising results [16,17].

Recently, there has been increasing interest in evaluating radiomic patterns of the region surrounding the visible tumor (peri-tumoral (PT)) and within the tumor habitat. For instance, radiomic analysis of the PT regions around lung nodules was shown to distinguish granulomas from adenocarcinomas on chest CT scans [18] and also found to be predictive of response to chemotherapy for lung [19] and breast cancers [20].

PCa is known to influence its surrounding habitat [16], resulting in areas with variable perfusion and permeability [21]. While evidence regarding the field effect of PCa on its habitat exists [16,22,23], surprisingly, there has been no work on the use of radiomics to characterize the peri-tumoral region surrounding MRI-visible lesions from the perspective of PCa risk stratification.

In this work, we evaluated the role of PT radiomics associated with heterogeneity patterns around the tumor as predictors of PCa risk categories defined by DRCS (1) independently, and (2) when combined with IT radiomics, using bi-parametric MRI (bpMRI). This work also represents one of the few that explicitly evaluates the role of radiomics for PCa risk stratification in a multi-site setting.

## 2. Results

### 2.1. Experiment 1: Using bpmri-Derived Peri-Tumoral Radiomic Features to Stratify PCa Risk as Defined by DRCS

**Figure 1:**
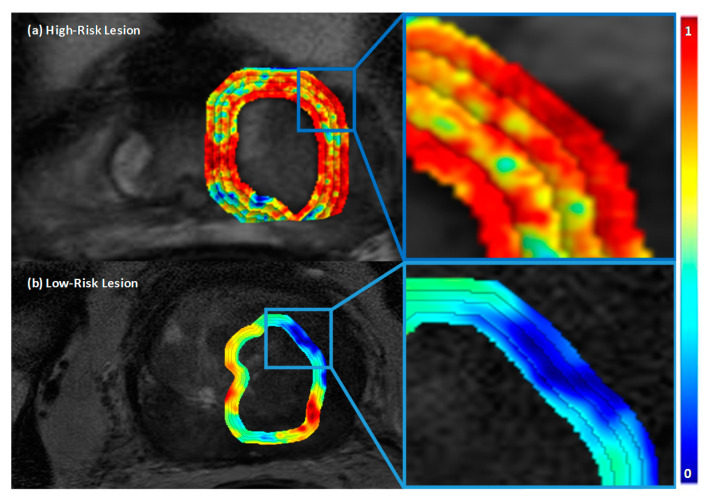
T2W MRI of a high risk and a low risk lesions (**left**) with their corresponding CoLlAGe entropy heat maps overlaid on the peri-tumoral (0–12 mm) regions (**right**, inset).

In this experiment, we evaluate the capability of IT and PT features individually in stratifying PCa risk as defined by DRCS. Table 1 lists the top 10 ranked radiomic features derived from prostate bpMRI selected by MRMR using the training cohort, D1, to stratify PCa risk in the Low-versus-High (L-vs.-H), and Low-versus-All (L-vs.-I + H) settings. First-order statistics, Gabor, and Haralick IT features; Gabor, Laws’ energy, and Haralick PT features from the 3–6 mm and 9–12 mm rings were found to be the most discriminating features. Figure 1 illustrates examples of high-risk and low-risk PCa lesions with differentially expressed PT radiomic features on T2W MRI.

For IT features, Receiver Operating Characteristic (ROC) analysis on the training cohort D1 yielded AUCs of 0.74, 0.75, and 0.82 for T2W, ADC, and T2W + ADC, respectively, in the L-vs.-I + H setting. In the L-vs.-H setting, AUCs were 0.79, 0.80, and 0.87, for T2W, ADC, and T2W + ADC, respectively. For the validation cohort D2, in the L-vs.-I + H setting, ROC analysis resulted in AUCs of 0.69, 0.71, and 0.75 for T2W, ADC, and T2W + ADC, respectively. Similarly, in the L-vs.-H setting, AUCs were 0.71, 0.79, and 0.81 for T2W and ADC, and T2W + ADC, respectively.

**Figure 2:**
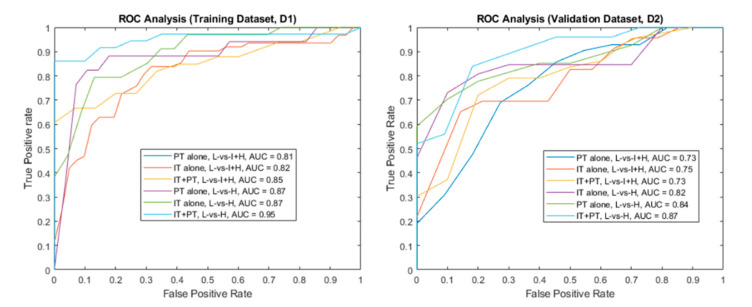
Receiver Operating Characteristic (ROC) analysis of Radiomic features derived from bi-parametric MRI for training (**left,** *n =* 151) and validation (**right,** *n =* 150) cohorts. AUCs increase significantly when intra-tumoral features are complemented with peri-tumoral features. AUCs of L-vs.-H are generally higher than those of L-vs.-I + H settings.

Figure 2 illustrates the ROC analysis results for PT features. The training cohort D1 yielded AUCs of 0.72, 0.70, and 0.81 for T2W, ADC, and T2W + ADC, respectively, in the L-vs.-I + H setting. In the L-vs.-H setting, AUCs were 0.73, 0.71, and 0.87, for T2W, ADC, and T2W + ADC, respectively. For the validation cohort D2, in the L-vs.-I + H setting, ROC analysis resulted in AUCs of 0.67, 0.68, and 0.73 for T2W, ADC, and T2W + ADC, respectively. Similarly, in the L-vs.-H setting, AUCs were 0.71, 0.76, and 0.84 for T2W and ADC, and T2W + ADC, respectively.

### 2.2. Experiment 2: Combining bpmri-Derived IT and PT Radiomics to Stratify PCa Risk as Defined by DRCS

The goal of this experiment was to evaluate the effect of combining IT and PT features in stratifying PCa risk. The best combination of IT and PT features was identified by MRMR and used to train a QDA classifier on D1 and then evaluated via ROC analysis on D2.

For D1, the best combination of IT and PT bpMRI features resulted in AUCs of 0.85 and 0.95 for the L-vs.-I + H and L-vs.-H classifications, respectively. Similarly, for D2, AUCs were 0.75 and 0.87 for the L-vs.-I + H and L-vs.-H settings, respectively.

Table 1 lists the top combined (IT and PT) radiomic features found to be most associated with PCa risk. A detailed list of the top performing radiomic features from the T2W and ADC sequences for intra-tumoral and individual peri-tumoral rings is provided in the (supplementary material Tables S1 and S2).

### 2.3. Experiment 3: Comparing Radiomics-Based Risk Stratification to PI-RADS

The goal of this experiment was to compare radiomics to PI-RADS v2 for PCa risk stratification. On bpMRI, based on PI-RADS scores, PCa lesions within D2 (*n* = 150 lesion, *N =* 115 patients) were partitioned into two groups of low (PI-RADS 1, 2) and high (PI-RADS 3–5) likelihood of clinically significant PCa.

Out of the 150 lesions evaluated, PI-RADS correctly identified 41 high-risk and 31 low-risk lesions (i.e., accuracy of 77.4% and 67.4%, respectively), while the radiomic model identified 37 high-risk and 42 low-risk lesions (i.e., accuracy of 69.8% and 91.3%, respectively) (Table 2).

## 3. Discussion

Since current standard-of-care for prostate cancer (PCa) characterization and monitoring continues to require frequent biopsies, there has been increasing interest in finding new ways for reliable non-invasive estimation of PCa risk [3,4,5], especially using MRI-derived radiomic texture analysis [11,12]. While a number of radiomic approaches have been previously presented for non-invasive risk characterization [9,10] and for predicting Gleason and tumor stage of PCa [12,13], these have typically employed only intra-tumoral (IT) radiomic texture features.

In this study, we evaluated the role of peri-tumoral (PT) radiomic texture features derived from bpMRI for PCa risk stratification. We demonstrated the value of IT and PT radiomics independently and when combined together for PCa risk stratification as defined by D’Amico Risk Classification System (DRCS). In concordance with previous studies [18,19,20], PT features were independently associated with PCa risk, however, we observed that they significantly improved the machine learning model’s predictive power when combined with IT features. Specifically, Haralick (3–6, 6–9 mm) and CoLlAGe texture features (6–9 mm) were observed to be over- and under-expressed, respectively, in high-risk compared to low- and intermediate-risk lesions.

When machine learning models were constructed based on radiomics from either T2W or ADC alone, PT features appeared to be generally more important on T2W MRI compared to ADC maps. ADC maps are derived from DWI which reflect mobility of water molecules around lesions, which is impacted by various factors such as cell density, membrane integrity, and microstructure heterogeneity. This is not the case with T2W MRI, which is more descriptive of the lesion anatomy and its surroundings.

In comparing PI-RADS v2 scores against predictions obtained from our combined IT and PT radiomic models, high-risk lesions were found to be associated with a high PI-RADS score, however the associations with low-risk lesions were not as clear. Interestingly, the combined IT + PT radiomic models appeared to perform better in identifying low-risk lesions, while performing comparable to PI-RADS in identifying high-risk lesions. These results are in alignment with previous studies where Chen et al. [24] found radiomics-based models outperforming PI-RADS for PCa detection and characterization. Similarly, Algohary et al. [17] found that PI-RADS did not always appear to corroborate with biopsy findings and IT-based radiomic models were found to outperform PI-RADS for identifying both clinically significant and non-significant PCa.

Our study was unique from some of these previously published PCa radiomic studies [17,24] in the following few ways. Firstly, this was the first study to demonstrate the added-value of PT radiomics when combined with IT radiomics for PCa risk stratification. Secondly, this is one of the few studies [14,25] to evaluate the role of radiomics from bpMRI across multiple sites (four sites in this study), most previous studies have been limited to a single site and thus have not had to deal with the issue of variance in acquisition parameters.

**Figure 3:**
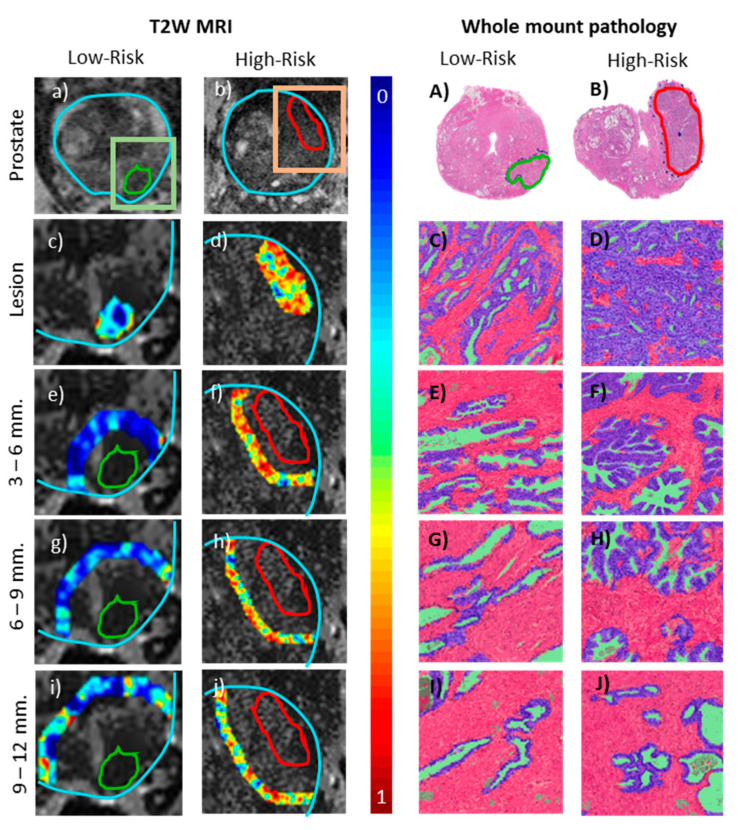
Peri-tumoral and intra-tumoral regions of interest (ROIs) overlaid with representative radiomic features on T2W MRI showing differential expression between low (**a**) and high (**b**) D’Amico risk prostate cancer patients, along with corresponding whole mount histopathology (**A**) and (**B**). Gabor feature map within the lesion (**c**) and (**d**) and representative pathology from corresponding region (**C** and **D**) with epithelium (purple), lumen (green) and stroma (pink) segmented. Similarly, Haralick (3–6 mm) (**e**) and (**f**), CoLlAGe (6–9 mm) (**g**) and (**h**) and Haralick (9–12 mm) (**i**) and (**j**) radiomic features within peri-tumoral annular rings within the prostate boundary (cyan). Representative peri-tumoral tissue compartments segmented on corresponding pathology are shown in (**E**–**J**). Quantitative feature values are provided in Supplementary Table S3.

A preliminary proof-of-concept experiment exploring the tissue compartments of epithelium, lumen and stroma within intra- and peri-tumoral regions on whole mount histopathology showed higher density of epithelium within and surrounding the high-risk PCa lesion. In Figure 3, we observe that radiomic features are overexpressed within the high-risk lesion which may reflect restricted diffusion due to collapsed gland structure. In addition, within the peri-tumoral region of high-risk lesions, a relatively higher concentration of epithelial cells and lymphocytes (blue) were identified, in turn suggesting an immune response to cancer. This may be reflected in terms of higher heterogeneity observed on T2W MRI which is reflected in terms of overexpression of peri-tumoral radiomic features compared to low-risk lesions. (Figure 3). These findings also appear to corroborate with previous studies where the density of stromal macrophages around the tumor was shown to be associated with likelihood of metastasis [26]. We acknowledge that these are very preliminary findings on a small cohort and further studies on larger datasets are warranted to validate these initial findings. However, differences observed in tissue pathology being reflected in peri-tumoral radiomics suggests that potentially discriminating information might exist in the peri-tumoral region on bpMRI.

Our study did have its limitations. First, PI-RADS 3 cases were included as part of the high PI-RADS group. This was done because there is no consensus on interpretation of these intermediate cases and in order to evaluate the ability of the radiomic features to distinguish the truly low-risk-low PI-RADS cases. A second limitation of this study was the small validation cohort size (*n* = 115 patients). Also, a small number of patients (*n* = 3) was used to explore association of PT radiomic features with histopathologic attribute. Third, inter-reader variations in delineation of PCa lesions and its sensitivity to machine learning was not evaluated. However, given the significant experience of radiologists and PI-RADS v2 guidelines [27], we expect this variation to be low and will be evaluating this in our future work. Fourth, for a high-risk patient, one of the lesions (in the case of multi-focal disease) could have been high-risk while the other would be low-risk. Unfortunately, we did not have that biopsy confirmation of every multi-focal lesion, only a per-patient level categorization of high-, intermediate- or low-risk disease. Future research directions may include analyzing intra and peri-tumoral radiomic features within respective zones (central zone (CZ), transition zone (TZ), peripheral zone (PZ)). Studies from our group [14,28] and others have established that the prostate cancer appearance is different between the PZ and TZ. Explicitly accounting for the geographical location of the tumor might allow for creation of zone-specific radiomic classifiers. In addition, another area for exploration would be the ability of the new radiomic features to predict outcome, not just D’Amico risk criteria. Towards that end, we would need to ensure that we have long-term outcome information available for the patients, this information was not available for the cohort we considered in this study.

## 4. Materials and Methods

Our study is HIPAA-compliant and IRB-approved, where a retrospective chart review with de-identified data was used and no protected health information was needed. Thus, need for an informed consent from all patients was waived.

### 4.1. Dataset Description

Between January 2007 and December 2015, biopsy-confirmed PCa lesions of patients from 4 independent institutions were retrospectively analyzed. Patients underwent 3-Tesla mpMRI scans and systematic 12-core TRUS-guided biopsies. Inclusion criteria included (a) availability of histopathology reports, (b) availability of all parameters to estimate the D’Amico risk for each PCa lesion of patient, and (c) presence of a screening or diagnostic MRI scan in the axial view. Table 3 illustrates the dataset and the number of lesions corresponding to each site.

**Figure 4:**
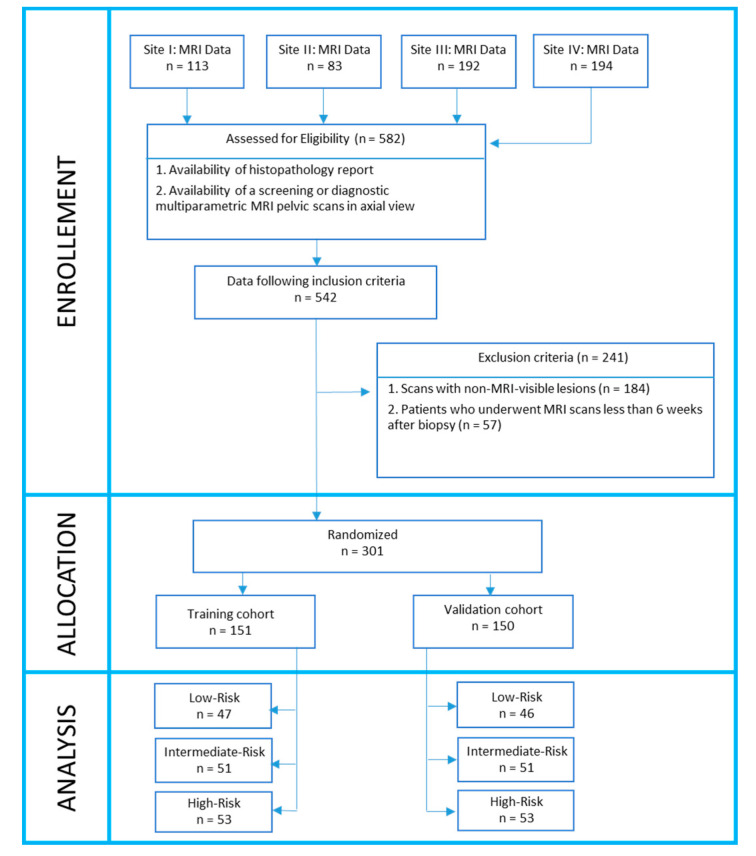
Dataset description. A 231-case dataset leading to 301 prostate cancer lesions was divided into 2 groups. Lesions from Group 1 were used to train machine learning classifiers. Lesions from Group 2 were used for independent validation.

To this cohort, we applied the following exclusion criteria: (1) scans with non-MRI-visible lesions (identified using histopathology reports), (2) patients who underwent MRI scans less than 6 weeks after biopsy in order to avoid hemorrhage artifacts in images. The final cohort comprised *n =* 301 lesions from *N =* 231 patients (Figure 4).

Additionally, for studying the association between PT radiomic features and histopathological attributes on whole mount histopathology, a preliminary proof-of-concept study with a subset of 3 patients who underwent radical prostatectomy (Institution 3), one each belonging to low-, intermediate-, and high-risk DRCS categories, was performed.

### 4.2. Lesion Delineation

Lesion regions of interest (ROIs) were delineated by expert radiologists at respective institutions (R1, R2, R3, R4: 7, 15, 15, and 25 years of experience in genitourinary radiology) on T2W MRI and Apparent Diffusion Coefficient (ADC) maps (derived from DWI) using 3D Slicer software v 4.10 (Kitware Inc., Carrboro, NC, USA). To minimize bias, radiologists were only provided positive core locations from the biopsy reports without any information regarding the pathologic findings. Radiologists assigned PI-RADS v2 scores to individual lesions. The lesion segmentations were then subsequently used to derive 4 annular peri-tumoral rings of 3 mm each, out to a maximum distance of 12 mm from the lesion boundary within the prostate. This was done in a way that was consistent with previous studies analyzing the tumor environment [29,30,31].

### 4.3. Pre-Processing

Each axial image from T2W MRI and ADC maps was resampled to a uniform pixel size of 0.5 × 0.5 mm^2^. Then, they were cropped to the lesion ROIs (with 2 mm padding) using the available prostate masks which were semi-automatically segmented by the radiologists. All the scans were interpolated to have 3 mm slice thickness to account for resolution differences during acquisition and were visually verified to ensure they occupy the same 3D space. Inherent scanner variability refers to the inherent drift between different MRI acquisitions, which causes image intensity values to lack tissue-specific meaning between studies. All scans were corrected for inherent scanner variability using a previously presented drift correction algorithm [32]. Studies acquired using an endo-rectal coil were bias-field-corrected using a previously published correction method [33].

### 4.4. Radiomic Feature Extraction

A total of 150 two-dimensional (2D) radiomic texture features (including first-order statistics, statistical, gray-level co-occurrence, steerable Gabor, co-occurrence of local anisotropic gradient orientations (CoLlAGe [34]), and Laws’ energy [35]) were extracted for from T2W images and ADC maps within each delineated PCa ROI (IT) and the peri-tumoral ROI (PT). A summary of the radiomic features and their significance in characterizing PCa is provided in Table 4. Feature extraction was performed in MATLAB (Mathworks, Inc., Natick, MA, USA).

**Figure 5:**
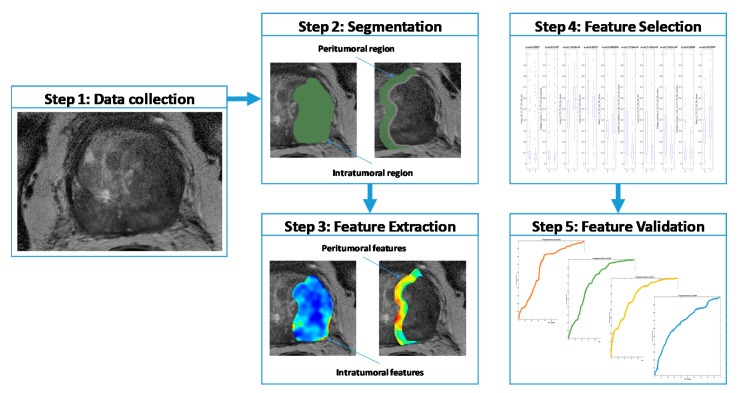
Flowchart illustrating our methodology. Bi-parametric MRI was retrospectively collected. Regions of interest were manually segmented in axial view to obtain intra-tumoral masks. Peri-tumoral masks were automatically generated for varying distances (shown here at 0–12 mm) outside the tumor. Haralick, Laws energy, CoLlAGe and Gabor texture features were extracted from tumor slices. Next, Wilcoxon rank-sum test and minimum-redundancy-maximum-relevance (MRMR) were implemented to select the top 10 features to train quadratic discriminant analysis classifiers and validate results on an independent dataset (D2, *n =* 150 lesions, *N =* 115 patients).

First-order statistics (mean, standard deviation, skewness, and kurtosis) of radiomic features were computed for the IT and PT ROIs and were normalized. In all, 300 IT radiomic features (150 from each of T2W and ADC) and, similarly, 1200 features from 4 PT ROIs of 3-mm-radius increments were extracted (Figure 5).

### 4.5. Association with Peri-Tumoral Histopathology

Whole-mounted prostatectomy (WMP) specimens obtained from 3 patients belonging to each of low-, intermediate-, and high-risk categories were stained with hematoxylin and eosin (H&E), sliced, and digitized at 20× magnification. Correspondences between mpMRI and WMP were obtained based off anatomical landmarks. An experienced pathologist delineated PCa ROI’s on digitized WMP. PT ROIs were obtained on bpMRI and WMP by computing annular rings within the prostate extending beyond the tumor ROI as previously described. Deep learning-based tissue segmentation approaches [36] were used to segment tissue compartments (epithelium, lumen and stroma) within the PT ROIs on WMP.

### 4.6. Statistical Analysis

Statistical analysis reported in our study was performed using MATLAB R2020A. The minimum-redundancy-maximum-relevance (MRMR) [37] feature selection algorithm was used to identify and rank order the top 10 radiomic features that differentiate low-risk from high-risk and from all lesions within the training set, D1, with an unadjusted *p*-value < 0.01 (using two-sided Wilcoxon rank sum tests) indicating statistical significance. This was done to avoid the curse of dimensionality, an issue where the number of features exceeds the number of patient studies, resulting in overfitting of the classification model.

Based on DRCS, lesions were categorized into 3 risk groups: low-, intermediate, and high-risk. The multi-institutional patient cohort was divided into two subsets: (1) training (D1, *n =* 151 lesions, *N =* 116 patients) and (2) independent hold-out validation (D2, *n =* 150 lesions, *N =* 115 patients).

A Quadratic Discriminant Analysis (QDA) machine-learning classifier in conjunction with feature selection was trained using D1 with a 100-run, 3-fold cross-validation (D1 was split into 3 random subsets, 2 were used for training and one for testing, the entire training-testing process was repeated 100 times), to distinguish the DRCS risk categories. The trained classifiers were validated using D2 in terms of area under the receiver operating characteristics curve (AUC). Multi-lesion cases were constrained to only be within one subset, i.e., all lesions from a single patient were strictly either used for training or validation but never for both.

## 5. Conclusions

In this multi-institutional study, we demonstrated that PT features are associated with a high degree of risk of PCa lesions on bpMRI, especially on T2W MRI. With additional independent validation, the combination of PT + IT radiomic features on prostate bpMRI could allow for non-invasive risk stratification and patient selection for active surveillance.

## Figures and Tables

**Table 1:** Results: The top 10 features from bi-parametric MRI for (a) Experiment 1: Intra-tumoral features alone, (b) Experiment 2: Peri-tumoral features alone, (c) Experiment 3: Intra-tumoral and Peri-tumoral features; in Low-versus-High, and Low-versus-(Intermediate + High) settings (*p*-values < 0.01).

	Low-vs.-(Intermediate + High)		Low-vs.-High	
**Experiment 1 (Intra-Tumoral features)**	**Feature Name (Parameters)**	**Protocol**	**Feature Name (Parameters)**	**Protocol**
	Mean (1)	ADC	Gabor (3, θ = 2.9 rad)	T2W
	Gabor (3, θ = 0.0 rad)	T2W	Mean (3)	T2W
	Mean (2)	ADC	Haralick (Sum of Average)	ADC
	Haralick (Sum of Average)	ADC	Mean (1)	ADC
	Variance (2)	ADC	Gabor (5, θ = 0.0 rad)	ADC
	Gabor (λ = 5, θ = 0.0 rad)	ADC	Gabor (3, θ = 0.1 rad)	ADC
	Gabor (λ = 4, θ = 0.0)	ADC	Gabor (3, θ = 0.7 rad)	T2W
	Gabor (λ = 3, θ = 0.1 rad)	ADC	Gabor (3, θ = 1.8 rad)	ADC
	Gabor (λ = 3, θ = 1.8 rad)	T2W	Gabor (3, θ = 2.4 rad)	ADC
	Gabor (λ = 3, θ = 2.4 rad)	ADC	Mean (2)	ADC
**Experiment 2 (Peri-Tumoral features)**	Haralick (Entropy difference) (3–6 mm)	T2W	Haralick (Info measure 1) (3–6 mm)	T2W
	Haralick (Momentum difference) (6–9 mm)	ADC	Haralick (Sum of Entropy) (3–6 mm)	ADC
	Gabor (lambda = 3, theta = 0 rad) (9–12 mm)	T2W	Haralick (Correlation) (3–6 mm)	ADC
	Haralick (Sum of Entropy) (3–6 mm)	T2W	Laws 9 (9–12 mm)	ADC
	Haralick (Entropy difference) (3–6 mm)	ADC	Laws (12) (3–6 mm)	T2W
	Haralick (Correlation) (3–6 mm)	ADC	Haralick (Info measure 2) (3–6 mm)	T2W
	Haralick (Entropy difference) (6–9 mm)	ADC	Haralick (Entropy) (3–6 mm)	ADC
	Gabor (λ = 3, θ = 0 rad) (6–9 mm)	ADC	Laws (11) (9–12 mm)	ADC
	Haralick (Info measure 2) (9–12 mm)	ADC	Laws (4) (9–12 mm)	ADC
	Haralick (Entropy difference) (6–9 mm)	T2W	Haralick (Energy)	ADC
**Experiment 3 (Intra- and Peri-Tumoral features)**	Laws (15)	T2W	Gabor (6 Hz, 2.0 rad) (3–6 mm)	T2W
	Canny	T2W	Gabor (6 Hz, 2.8 rad) (3–6 mm)	T2W
	Collage (Entropy) (6–9 mm)	ADC	Haralick (Momentum Sum)	ADC
	Laws (11)	ADC	Gabor (6 Hz, 1.8 rad)	ADC
	Haralick (Entropy)	ADC	Mean (9–12 mm)	T2W
	Collage	ADC	Gabor (2.5 Hz, 0.4 rad)	T2W
	Haralick (Info measure 1) (3–6 mm)	T2W	Gabor (3 Hz, 0.4 rad)	T2W
	Laws (17) (3–6 mm)	ADC	Gabor (3.5 Hz, 0.4 rad)	T2W
	Haralick (Info measure 2)	T2W	Gabor (5 Hz, 1.6 rad)	ADC
	Haralick (Info measure 2)	ADC	Gabor (6 Hz, 1.6 rad)	ADC

**Table 2:** Risk stratification results of PCa lesions for PI-RADS v2 and radiomics, based on D’Amico Risk Classification System (DRCS) criteria (Low-versus-(Intermediate + High) setting).

D’Amico Classification	PI-RADS v2		Total	Combined Radiomic Features (IT + PT)	
	High (3–5)	Low (1–2)		High	Low
**High-Risk**	41	12	53	37	16
**Intermediate-Risk**	33	18	51	28	23
**Low-Risk**	15	31	46	4	42
**Total**	89	61	150	69	81

**Table 3:** Dataset description.

**Cohort**	**Institution 1**	**Institution 2**	**Institution 3**	**Institution 4**
Number of Subjects	32	73	45	81
Age (mean ± SD)	65.1 ± 6.4	62.6 ± 10.8	64.3 ± 5.6	68.5 ± 8.05
PSA (mean ± SD) ng/mL	6.9 ± 5.8	5.9 ± 4.2	9.8 ± 6.3	8.08 ± 6.1
Lesion size (mean ± SD) cm^3^	1.10 ± 1.79	0.67 ± 0.82	1.02 ± 1.16	0.86 ± 0.66
Gleason Scores (number of lesions)	6(8), 7(8), 8(11), 9(5)	6(23), 7(8), 8(9), 9(33)	6(8), 7(11), 8(16), 9(10)	6(38), 7(24), 8(13), 9(6)
PI-RADS (mean ± SD)	4.19 ± 1.05	3.65 ± 1.06	3.59 ± 1.35	2.56 ± 1.59
**Scanner**				
Manufacturer	Philips Achieva	Siemens Verio	Siemens Verio	Philips Achieva
Coil type	Body coil	Endorectal coil	Body coil	Endorectal coil
**T2-Weighted MRI**				
Field-of-view (mm^2^)	220 × 220	140 × 140	200 × 200	260 × 260
Matrix size	444 × 332	384 × 384	320 × 320	256 × 256
**Diffusion-Weighted MRI**				
Field-of-view (mm^2^)	180 × 180	260 × 186	260 × 260	260 × 260
Matrix size	128 × 128	116 × 162	128 × 128	128 × 128
b-values (s/mm^2^)	0, 1500	0, 50, 1000, 1500, 2000	0, 50, 600, 1000, 1400	0, 400, 900, 1500

**Table 4:** Description of Radiomic features extracted.

Feature Category	Feature Type	Number of Features Extracted (Total)	Relevance to Prostate Cancer
Signal Intensity	T2w images, ADC maps	1 × 2 (2)	Cancers are usually hypo-intense on MRI
First Order Statistics	Mean, Median, Sobel	9 × 2 (18)	Intensity variability
Gabor	Frequency, Orientation	76 × 2 (152)	Low-level oriented edges
Gray-level co-occurrence	Haralick	3 × 13 × 2 (78)	Structural heterogeneity
Texture Energy	Laws’ texture energy	25 × 2 (50)	Appearance of ROI

## Supplementary Materials

The following are available online at https://www.mdpi.com/2072-6694/12/8/2200/s1. Table S1. Top 10 Intra- and Peri-tumoral radiomic features from T2-weighted images and ADC maps, in Low-vs.-High setting (D1, *n* = 151); Table S2. Top 10 Intra- and Peri-tumoral radiomic features from T2-weighted images and ADC maps, in Low-vs.-(Intermediate + High) setting (D1, *n* = 151); Table S3. Radiomic features in various peri-tumoral regions of the prostate along with corresponding percentage of epithelium, lumen, and stroma on histopathology for low-, intermediate-, and high-risk patients as defined by D’Amico risk classification system.
