# Supplementary material for: Combination of Peri-Tumoral and Intra-Tumoral Radiomic Features on Bi-Parametric MRI Accurately Stratifies Prostate Cancer Risk: A Multi-Site Study
Source: Cancers (Basel). 2020 Aug 6;12(8):2200. doi: 10.3390/cancers12082200 (PMC7465024; doi:10.3390/cancers12082200)
Supplement: Supplementary file 1 [file cancers-12-02200-s001.pdf]

Supplementary Materials

Combination of Peri-Tumoral and Intra-Tumoral Radiomic Features on Bi-Parametric MRI Accurately Stratifies Prostate Cancer Risk: A Multi-Site Study

Ahmad Algohary, Rakesh Shiradkar, Shivani Pahwa, Andrei Purysko, Sadhna Verma, Daniel Moses, Ronald Shnier, Anne-Maree Haynes, Warick Delprado, James Thompson, Sreeharsha Tirumani, Amr Mahran, Ardeshir R Rastinehad, Lee Ponsky, Phillip D. Stricker and Anant Madabhushi

Table S1. Top 10 Intra- and Peri-tumoral radiomic features from T2-weighted images and ADC maps, in Low-vs.-High setting (D1, n = 151).

L-H	Intra-tumoral	Peri-Tumoral				
		0–3 mm	3–6 mm	6–9 mm	9–12 mm	All
ADC	Gabor 3 0 kurtosis	Gabor 5 0 mean	Haralick SumAve 1 64 mean	Haralick SumAve 1 64 mean	Range 2 kurtosis	Laws 7 kurtosis
	Haralick SumAve 1 64 mean	Haralick SumAve 1 64 skewness	Haralick SumAve 1 64 skewness	Laws 24 std	Haralick SumAve 1 64 skewness	Haralick SumAve 1 64 mean
	Haralick SumVar 1 64 skewness	Haralick SumAve 1 64 mean	Gabor 5 0 skewness	Range 2 kurtosis	Haralick Energy 1 64 kurtosis	Gabor 5 0 mean
	Gabor 5 0 mean	Mean 1 skewness	Mean 1 skewness	Haralick Energy 1 64 std	Laws 2 kurtosis	Haralick DiffAve 1 64 kurtosis
	Laws 7 kurtosis	Gabor 5 mean	Gabor 3 0 skewness	Haralick Intertia 1 64 kurtosis	Laws 4 kurtosis	Gabor 5 0 kurtosis
	Mean 1 skewness	Laws 1 mean	Gabor 5 skewness	Haralick SumAve 1 64 skewness	Laws 21 std	Haralick SumAve 1 64 mean
	Haralick DiffVar 1 64 kurtosis	Laws 1 skewness	Laws 1 skewness	Laws 4 kurtosis	Haralick Intertia 1 64 mean	Gabor 5 0 skewness
	Laws 2 kurtosis	Gabor 5 1p mean	Gabor 5 1p skewness	Haralick SumVar 1 64 kurtosis	Range 1 kurtosis	Laws 6 kurtosis
	Haralick Energy 1 64 std	Gabor 5 18 mean	Gabor 3 skewness	Laws 6 kurtosis	Laws 5 std	Laws 2 kurtosis
T2	Gabor 3 kurtosis	Gabor 5 0 skewness	Gabor 5 18 skewness	Haralick Entropy 1 64 std	Haralick SumEnt 1 64 std	Mean 1 kurtosis
	Mean 2 skewness	Mean 2 mean	Laws 19 mean	Haralick Correlation 1 64 mean	Mean 2 skewness	Gabor 3 0 skewness
	Haralick SumEnt 1 64 std	Collage DiffVar 1 skewness	Haralick Correlation 1 64 mean	Haralick DiffEnt 1 64 skewness	Collage DiffVar 1 kurtosis	Haralick SumVar 1 64 kurtosis
	Gabor 5 0 mean	Laws 25 std	Haralick DiffEnt 1 64 skewness	Laws 3 mean	Haralick Correlation 1 64 mean	Mean 1 kurtosis
	Laws 6 kurtosis	Haralick Intertia 1 64 skewness	Collage SumVar 1 std	Haralick SumAve 1 64 mean	Laws 5 skewness	Haralick SumAve 1 64 mean

Gabor 5 0 kurtosis	Collage DiffVar 1 mean	Haralick Correlation 1 64 std	Haralick InDiffMom 1 64 mean	Haralick SumAve 1 64 skewness	Gabor 5 0 mean
Haralick Entropy 1 64 std	Collage DiffAve 1 kurtosis	Haralick Correlation 1 64 skewness	Haralick Correlation 1 64 skewness	Laws 13 skewness	Gabor 5 0 kurtosis
Gabor 3 0 skewness	Haralick Correlation 1 64 mean	Haralick InDiffMom 1 64 mean	LoG kurtosis	Haralick Correlation 1 64 std	Mean 2 skewness
Gabor 5 mean	Gabor 5 0 mean	Collage Intertia 1 std	Haralick Entropy 1 64 kurtosis	Mean 1 skewness	Laws 6 kurtosis
Gabor 3 skewness	Collage Intertia 1 mean	Collage DiffVar 1 std	Range 2 skewness	Haralick Correlation 1 64 skewness	Haralick SumEnt 1 64 skewness
Gabor 5 kurtosis	Haralick DiffVar 1 64 kurtosis	Haralick InDiffMom 1 64 skewness	Haralick Entropy 1 64 skewness	Gabor 3 0 skewness	Gabor 3 skewness

Table S2. Top 10 Intra- and Peri-tumoral radiomic features from T2-weighted images and ADC maps, in Low-vs.-(Intermediate + High) setting (D1, n = 151).

L-A	Intra-tumoral	Peri-Tumoral				
		0–3 mm	3–6 mm	6–9 mm	9–12 mm	All
ADC	Mean 1 mean	Mean 1 mean	Collage DiffEnt 1 mean	Laws 21 std	Laws 22 std	Laws 1 skewness
	Gabor 3 0 skewness	Collage DiffVar 1 std	Haralick SumAve 1 64 mean	Laws 10 std	Collage Intertia 1 skewness	Mean 2 mean
	Mean 2 mean	Haralick SumAve 1 64 mean	Collage Entropy 1 std	Collage DiffEnt 1 mean	Laws 10 std	Gabor 3 0 skewness
	Haralick SumAve 1 64 mean	Collage DiffAve 1 std	Mean 2 skewness	Laws 15 std	Laws 16 std	Haralick SumAve 1 64 mean
	Mean 2 skewness	Laws 1 mean	Collage InDiffMom 1 std	Haralick Entropy 1 64 std	Collage DiffAve 1 mean	Mean 1 skewness
	Gabor 5 0 skewness	Mean 2 mean	Collage SumVar 1 mean	Laws 22 std	Laws 20 std	Mean 1 mean
	Gabor 3 skewness	Collage Intertia 1 std	Mean 1 mean	Laws 20 std	Canny skewness	Gabor 3 skewness
	Gabor 3 skewness	Gabor 3 0 mean	Collage SumEnt 1 mean	Laws 23 std	Laws 23 std	Gabor 3 skewness
	Gabor 3 18 skewness	Gabor 3 mean	Collage DiffAve 1 mean	Collage Intertia 1 mean	Laws 2 kurtosis	Gabor 3 18 skewness
	Gabor 3 24 skewness	Gabor 3 mean	Haralick SumAve 1 64 skewness	Laws 10 mean	Laws 18 std	Gabor 3 24 skewness
T2	Mean 2 mean	Mean 2 mean	Laws 22 std	Haralick Correlation 1 64 mean	Laws 24 std	Mean 2 mean
	Laws 1 kurtosis	Haralick Correlation 1 64 mean	Collage Intertia 1 kurtosis	Laws 9 mean	Mean 1 skewness	Mean 2 kurtosis
	Laws 6 kurtosis	Haralick Correlation 1 64 std	Laws 2 mean	Haralick Correlation 1 64 std	Collage DiffAve 1 skewness	Haralick Correlation 1 64 std
	Mean 1 mean	Haralick DiffVar 1 64 kurtosis	Laws 21 std	Haralick InDiffMom 1 64 skewness	Collage DiffVar 1 kurtosis	Laws 6 kurtosis
	Haralick DiffVar 1 64 kurtosis	Collage DiffAve 1 mean	Mean 1 mean	Laws 3 mean	Range 2 std	Laws 1 kurtosis
	Mean 2 kurtosis	Mean 1 mean	Haralick Correlation 1 64 mean	Collage DiffEnt 1 skewness	Haralick Correlation 1 64 mean	Mean 2 mean

Haralick DiffVar 1 64 skewness	Haralick Intertia 1 64 skewness	Laws 23 std	Haralick SumAve 1 64 mean	Laws 21 std	Haralick Intertia 1 64 kurtosis
Gabor 3 0 skewness	Collage SumVar 1 mean	Collage Intertia 1 mean	Haralick Correlation 1 64 skewness	Laws 5 skewness	Mean 1 mean
Gabor 3 0 kurtosis	Collage SumVar 1 kurtosis	Haralick Energy 1 64 kurtosis	Collage InfoMes2 1 kurtosis	Laws 22 std	Gabor 3 0 kurtosis
Haralick Intertia 1 64 kurtosis	Gabor 5 0 mean	Mean 2 mean	Haralick SumEnt 1 64 skewness	Haralick Correlation 1 64 std	Gabor 3 0 skewness

Table S3. Radiomic features in various peri-tumoral regions of the prostate along with corresponding percentage of epithelium, lumen, and stroma on histopathology for low-, intermediate-, and high-risk patients as defined by D'Amico risk classification system.

Region	**Low-risk**				**Intermediate-risk**				**High-risk**			
	**IT**	**PT (mm)**			**IT**	**PT (mm)**			**IT**	**PT (mm)**		
	Lesion	3–6	6–9	9–12	Lesion	3–6	6–9	9–12	Lesion	3–6	6–9	9–12
% epithelium	0.30	0.12	0.17	0.09	0.43	0.13	0.16	0.10	0.63	0.18	0.19	0.13
% lumen	0.20	0.18	0.19	0.20	0.17	0.14	0.21	0.24	0.09	0.29	0.27	0.23
% stroma	0.50	0.71	0.64	0.70	0.40	0.73	0.63	0.66	0.28	0.52	0.53	0.64
Gabor (T2w)	0.45				0.48				0.71			
Haralick Sum Entropy (T2w)		0.23				0.34				0.66		
CoLlAGe Entropy (T2w)			0.18				0.23				0.58	
Haralick Info Meas. (T2w)				0.32				0.31				0.63

© 2020 by the authors. Licensee MDPI, Basel, Switzerland. This article is an open access article distributed under the terms and conditions of the Creative Commons Attribution (CC BY) license (http://creativecommons.org/licenses/by/4.0/).
